# An In Vitro Orbital Flow Model to Study Mechanical Loading Effects on Osteoblasts

**DOI:** 10.3390/biology13090646

**Published:** 2024-08-23

**Authors:** Subburaman Mohan, Ritika Surisetty, Chandrasekhar Kesavan

**Affiliations:** 1 Musculoskeletal Disease Center, VA Loma Linda Healthcare System, Loma Linda, CA 92357, USA; subburaman.mohan@va.gov (S.M.); surisettyritika@gmail.com (R.S.); 2 Department of Medicine, Loma Linda University, Loma Linda, CA 92354, USA; 3 Orthopedic Surgery, Loma Linda University, Loma Linda, CA 92354, USA

**Keywords:** mechanical loading, orbital flow, osteoblasts, gene expression, differentiation

## Abstract

**Simple Summary:**

Models to study the effects of exercise-induced benefits on bone-forming osteoblasts are limited and expensive. Although an orbital shaker model is simple and inexpensive, the impact of the flow produced by an orbital shaker on osteoblasts is not well defined, and this was evaluated in this study using an in vitro model. The findings of this study show that the flow produced by orbital shaking at physiological levels stimulates anabolic effects on osteoblasts, as evident from an increase in cell number and expression levels of osteoblast differentiation markers and mechanosensitive genes. Therefore, an orbital shaker at lower frequency provides an inexpensive and appropriate model to study mechanical strain effects on osteoblasts in vitro.

**Abstract:**

Flow induced by an orbital shaker is known to produce shear stress and oscillatory flow, but the utility of this model for studying mechanical loading effects in osteoblasts is not well defined. To test this, osteoblasts derived from the long bones of adult male C57BL/6J mice were plated on 6-well plates and subjected to orbital shaking at various frequencies (0.7, 1.4, and 3.3 Hz) for 30 and 60 min in serum-free differentiation media. The shear stress on cells produced by 0.7, 1.4, and 3.3 Hz shaking frequencies were 1.6, 4.5, and 11.8 dynes/cm^2^, respectively. ALP activity measured 72 h after shaking (orbital flow) showed a significant increase at 0.7 and 1.4 Hz, but not at 3.3 Hz, compared to static controls. Orbital flow-induced mechanical stress also significantly increased (25%) osteoblast proliferation at a 0.7 Hz flow compared to static controls. Additionally, expression levels of bone formation markers *Osf2*, *Hif1a*, *Vegf*, and *Cox2* were significantly increased (1.5- to 3-fold, *p* < 0.05) in cells subjected to a 0.7 Hz flow compared to non-loaded control cells. We also evaluated the effect of orbital flow on key signaling pathways (mTOR, JNK, and WNT) known to mediate mechanical strain effects on osteoblasts. We found that blocking mTOR and WNT signaling with inhibitors significantly reduced (20–30%) orbital flow-induced ALP activity compared to cells treated using a vehicle. In contrast, inhibition of JNK signaling did not affect flow-induced osteoblast differentiation. In conclusion, our findings show that the flow produced by an orbital shaker at a lower frequency is an appropriate inexpensive model for studying the molecular pathways mediating mechanical strain effects on primary cultures of osteoblasts in vitro.

## 1. Introduction

Mechanical loading is an important regulator of skeletal growth and maintenance [1,2,3,4]. Previous studies have shown that loading stimulates new bone formation [2,5,6,7], while non-loading results in bone loss leading to pathological conditions like osteopenia and osteoporosis [8,9,10]. Thus, exercise has been used as a strategy to reduce or prevent bone loss [2,11,12]. However, in conditions where patients are bedridden [13] (spinal cord injury and aging), exercise is not feasible. Therefore, our studies have focused on understanding the mechanisms by which exercise regulates bone-forming osteoblast function in an effort to identify novel therapeutic targets whose regulation might stimulate new bone formation when exercise is not possible.

It is known from studies that bones are not loaded in a sinusoidal manner, but are loaded repetitively, inducing fluid flow through the lacunar/canalicular network, which is reversed, causing oscillatory flow [14]. To mimic this loading effect in vitro on bone cells, several loading models have been developed that include a fluid flow chamber and an oscillatory flow apparatus [5,14,15]. These models involve complex equipment setups and are relatively expensive. As the flow induced by an orbital shaker is also known to produce shear stress and oscillatory flow, this study evaluated the utility of this model for mechanical loading effects on osteoblasts.

## 2. Materials and Methods

### 2.1. Osteoblasts Cultures

Cells were isolated from marrow flushed long bones of adult C57BL/6J mice using collagenase digestion. These cells were cultured in alpha minimal essential medium (αMEM) containing 10% fetal bovine serum (FBS) and antibiotics (penicillin, 100 units/mL, and streptomycin, 100 µg/mL).

### 2.2. Orbital Shaker and Rocker Flow Models

First-passage cells (approximately 10,000/well) in a 300 µL volume of medium were plated on the peripheral region of 6-well plates. The plates were incubated for 20 min in a 5% CO_2_ incubator so the cells could attach. Subsequently, 2 mL of media containing serum and antibiotics were added to each plate, and incubation at 5% CO_2_ continued for 24 h. Media was replaced with serum-free media containing 0.1% bovine serum albumin (BSA), and twenty-four hours later cells were subjected to orbital shaking in serum-free media at speeds of 3, 4, and 5, which corresponded to 0.7, 1.4, and 3.3 frequencies (Hz), respectively, for 30 and 60 min. For the rocker model, cells were subjected to rocking using a see-saw rocker for 30 min, at speeds of 6 and 8, which corresponded to 0.2 and 0.28 Hz, respectively. For both models, no-flow plates (static controls) were kept inside the 5% CO_2_ incubator for the same time. Videos depicting the orbital shaker and see-saw rocker models used in this study are shown in the Supplementary Materials.

### 2.3. Osteoblast Proliferation Assay

First-passage cells were plated in 6-well plates and cultured in αMEM containing 10% CS and antibiotics for 24 h, followed by another 24 h in αMEM containing 0.1% BSA and antibiotics. Cells were subjected to mechanical stress produced by an orbital shaker or a see-saw rocker. The experiment was terminated 48 h after the flow cycle, and proliferation was assessed using a Cy-Quant Dye Kit (Life Technologies, Carlsbad, CA, USA) according to the manufacturer’s instructions.

### 2.4. Differentiation Assay

Alkaline phosphatase (ALP) activity was used as a measure of osteoblast differentiation. For the ALP assay, prior to in vitro mechanical loading, osteoblasts were cultured in serum containing media for 24 h, followed by 24 h in serum-free media. Next, the differentiation medium containing 0.1% BSA, ascorbic acid (100 µg/mL), and β-glycerophosphate (10 mM) was added to the plates, and then subjected to orbital flow. Seventy hours post flow, the experiment was terminated. Briefly, cells were washed with 1× PBS, and 100 µL of ALP lysis buffer was added. Cell lysates were stored at −80 °C. Twenty-four hours later, the ALP substrate was added to the lysate, and plates were read at 410–490 nm. The total protein was also measured from cell lysates using a bicinchoninic acid (BCA) assay, and ALP activity was quantitated as described [16].

Subsequently, orbital flow-induced ALP activity was assessed in the presence or absence of inhibitors of signaling pathways that are known to mediate mechanical loading effects. Prior to in vitro mechanical loading, the differentiation medium containing 0.1% BSA, ascorbic acid (100 µg/mL), and β-glycerophosphate (10 mM) was added to the plates. Inhibitors of either WNT (50 µM, JW74, Tocris, MN, USA), mTOR (200 mM, Rapamycin, Sigma-Aldrich, St. Louis, MO, USA), or JNK (1 µM, Inhibitor II, Millipore Sigma, Burlington, MA, USA) signaling were added, and plates were incubated for 1 h and then subjected to orbital shaking (orbital flow) for 60 min. After the flow treatment, plates were incubated at 5% CO_2_ for 72 h and then terminated to quantitate ALP activity by measuring para-nitrophenol (Sigma-Aldrich, St. Louis, MO, USA) using a plate reader [16].

### 2.5. Gene Expression

We isolated total ribonucleic acid (RNA) from cells subjected to flow and no-flow using a Qiagen isolation kit protocol as per the manufacturer’s instructions (Qiagen, Germantown, MD, USA). Two hundred nanograms of purified total RNA was used to synthesize the first strand complementary deoxyribose nuclei acid (cDNA) by reverse transcription as per the manufacturer’s instructions (Bio-Rad, Hercules, CA, USA). The first strand DNA was subjected to real-time PCR amplification using a SYBR green master mix and gene-specific primers (IDT DNA technology, San Diego, CA, USA) on a ViiA7 real-time PCR system (Applied Biosystems, Waltham, MA, USA). The endogenous control (β-*actin*) was used to normalize data, and normalized values were subjected to the 2^∆∆Ct^ formula (where C+ is the contraction threshold) to calculate the fold change between the vehicle and experimental groups [17].

### 2.6. Immunofluorescence Labeling of Cells

Cells on 6-well plates were subjected to orbital flow for 60 min and terminated 30 min after the flow experiment. The static control was kept inside the incubator without orbital flow. Cells were rinsed with 1× PBS and fixed with 2 mL of 4% paraformaldehyde for 15 min. Cells were rinsed three times with 1× PBS for 5 min, with gentle shaking, and then treated with 2 mL of 1% SDS to increase cellular permeability. Cells were washed with 1× PBS once and then blocked with 0.4% goat serum with the blocking buffer for 1 h. Cells were incubated with mTOR rabbit primary antibody (Cell Signaling, Danvers, MA, USA) or mouse β-catenin monoclonal antibody (Sigma, St. Louis, MO, USA), diluted 1:500 and 1:1000, respectively, in the antibody dilution buffer for 48 h at 4 °C, with gentle shaking. Forty-eight hours later, cells were washed with 1× PBS containing 0.01% Tween-20 three times for 5 min and incubated with Alexa 555 fluorochrome-conjugated secondary antibody and Alexa 488 fluorochrome-conjugated secondary antibody (mouse anti-goat, Molecular Probes, Eugene, OR, USA) diluted in antibody dilution buffer for 2 h while being protected from light, with gentle shaking. After 2 h, cells were rinsed with 1× PBS once, incubated with DAPI (Cell Signaling, Danvers, MA, USA) for nuclear staining, and rinsed three times for 5 min with 1× PBS. Cells were protected from light, and the fluorescent signal for key signaling proteins was visualized under 50× magnification using a Lecia fluorescence microscope.

### 2.7. Statistical Analysis

Data are presented as the mean ± SEM. The Student *t*-test was used to evaluate differences between flow-induced effects on cells compared to static controls. A *p*-value of <0.05 was considered statistically significant.

## 3. Results and Discussion

Multiple mechanical loading devices have been developed and tested to study the effects of mechanical strain on bone-forming osteoblast cell function. Among these models, rocker and orbital flow models are known to produce shear stress and oscillatory flow, respectively. As these two models are easy to set up and relatively inexpensive, we evaluated the mechanical strain effects produced using orbital flow and rocker models on osteoblast proliferation and ALP activity, which are well-established measures of osteoblast differentiation. Prior to subjecting osteoblasts to in vitro flow stress, we first assessed the flow output produced at different speeds by orbital and see-saw rocker models by adding 2 mL of medium into 6-well plates. We found that the flow induced by the orbital shaker at speeds 1 and 2 and by the see-saw rocker at speed 5 produced only 10–13 and 9 revolutions per minute (rpm), respectively. Because of the relatively slow oscillation speed, it was unlikely that there would be effects on cells in the short duration of loading using these speeds. Therefore, other rotational speeds in both models were assessed. In the orbital shaker model, at speed 3 the flow around cells was visible and 42 rpm was produced in the 6-well plate. In the see-saw model, the flow produced was 16–20 rpm (see-saw rocking) at speed settings 6 and 8 in the 6-well plate. The amount of mechanical strain (dyne/cm^2^) applied to cells in 6-well plates at speed 3 in the orbital shaker and at speeds 6 and 8 in the see-saw rocker model were determined using the formulas τ_max_ = a√ηp(2πf)^3^ and τ = π*µθ_max_*/2δ^2^*T*, respectively, as described [18]. In the formula τ_max_ = a√ηp(2πf)^3^, τ_max_ corresponds to shear stress expressed as dynes/cm^2^. The “a” is the orbital radius of the rotation of the orbital shaker, η is the viscosity of the culture medium, p is the density of the culture medium, and f is the frequency of the rotation (rotation/second). In the formula τ = π*µθ_max_*/2δ^2^*T*, τ represents shear stress at the bottom of the plate, *µ* is the fluid viscosity, *θ_max_* corresponds to the maximum flip angle, δ^2^ is the fluid depth to well length, and *T* is the time required for one cycle per second [19]. Using the above formula, in the orbital shaker we found that 42 rpm represented approximately 0.7 Hz, which corresponded to 1.61 dyne/cm^2^/min of shear stress applied to the cells. In the rocker model, we found that 12 and 17 rpm corresponded to 0.2 and 0.28 Hz, respectively, which corresponded to 0.03 and 0.043 dyne/cm^2^/min of shear stress applied to the cells, respectively. Based on these data, we subjected osteoblasts to orbital shaking at 0.7 Hz and to see-saw rocking at 0.2 and 0.28 Hz.

**Figure 1:**
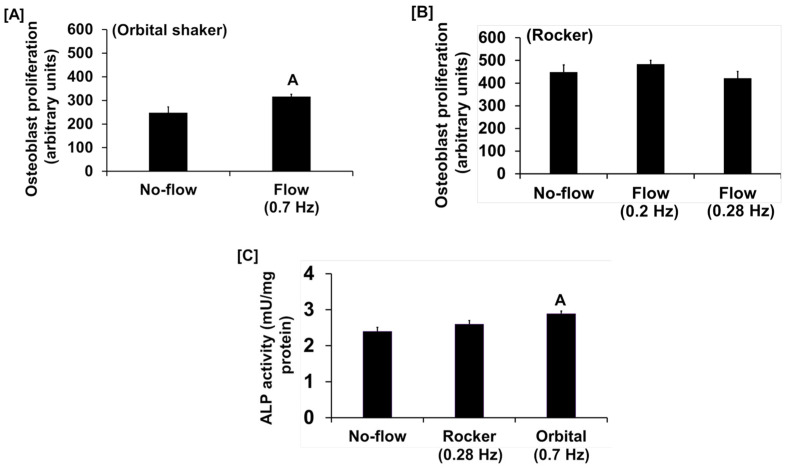
Proliferation response of cultured osteoblasts to (**A**) orbital shaking and (**B**) rocker shaking, and (**C**) ALP activity response to orbital and rocker shaking. Values are mean ± SEM, n = 8, and ^A^ *p* < 0.05 vs. no-flow.

**Figure 2:**
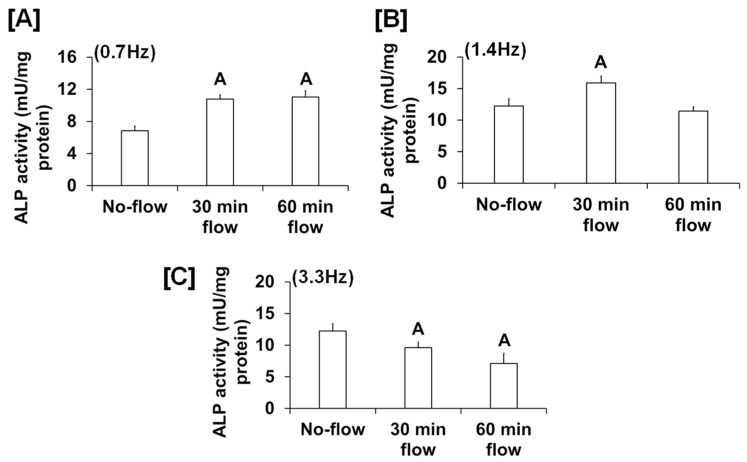
ALP activity in osteoblasts subjected to orbital flow stress at different frequencies: (**A**) 0.7 Hz, (**B**) 1.4 Hz, and (**C**) 3.3 Hz for 30 and 60 min durations. Values are mean ± SEM, n = 5, and ^A^ *p* < 0.05 vs. no-flow.

We found that the fluid flow generated by an orbital shaker moving at 0.7 Hz significantly increased osteoblast proliferation (Figure 1A), while this effect was not significant for the flow applied to cells at 0.2 Hz by the see-saw rocker model (Figure 1B). In addition to proliferation, we found that the orbital shaker model produced a significant increase in osteoblast differentiation, as measured by ALP activity compared to stationary osteoblasts (static control) (Figure 1C). However, there was no increase in osteoblast differentiation using the rocker model in response to an applied flow of 0.28 Hz. The most likely explanation for the lack of an ALP response in the rocker model could be that the amount of shear stress applied to the cells was too low, and a higher frequency of flow may be required to stimulate an anabolic effect on the cells. We next evaluated whether increasing the frequency and duration of orbital shaking maximized flow-induced anabolic effects on the cells. To test this, osteoblasts were subjected to the established speeds 3, 4, and 5, which produced 42, 84, and 198 rpm, respectively, in the 6-well plates, which corresponded to 0.7, 1.4, and 3.3 Hz and 1.6, 4.5, and 11.8 dyne/cm^2^/min shear stresses, respectively, on the cells. We found that the flow at 0.7 Hz for 30 and 60 min was anabolic, as represented by increased ALP activity (Figure 2A). However, for the flow applied at 1.4 Hz, this effect was observed only at 30 min, and not at 60 min (Figure 2B). Furthermore, in cells subjected to a flow of 3.3 Hz, the ALP activity was significantly decreased (Figure 2C). These data suggest that increasing the frequency of loading beyond 0.7 Hz in the orbital shaker did not increase osteoblast function.

**Figure 3:**
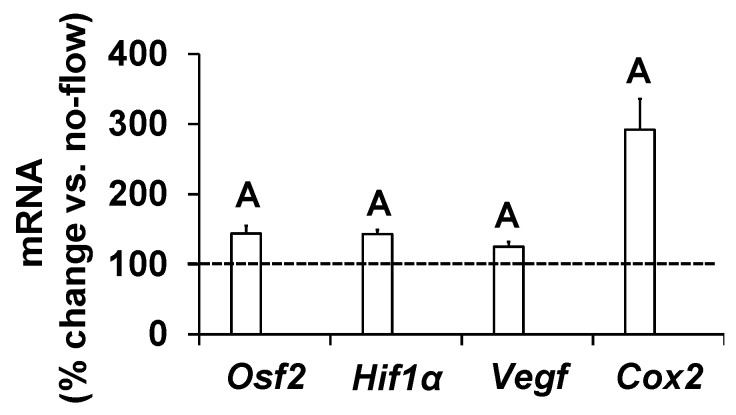
Expression levels of mechanosensitive genes in response to orbital flow in osteoblasts. Values are mean ± SEM, n = 5, and ^A^ *p* < 0.05 vs. no-flow.

To further determine if increased osteoblast activity in response to orbital shaking was the consequence of increased gene expression, we measured expression levels of genes that have been implicated as mediators of mechanical strain in osteoblasts [17,20,21,22]. Cells were exposed to an orbital flow of 0.7 Hz for 60 min, and the study was terminated after four hours. Total RNA was isolated, and cDNA was synthesized. Real-time PCR was used to quantitate the expression of genes in cells subjected to flow and no-flow. We found that the expression levels of *Osf2*, *Cox2*, *Vegf*, and *Hif1α* significantly increased in response to this orbital flow, demonstrating that the orbital shaker was effective at increasing the expression of genes involved in promoting bone formation (Figure 3).

**Figure 4:**
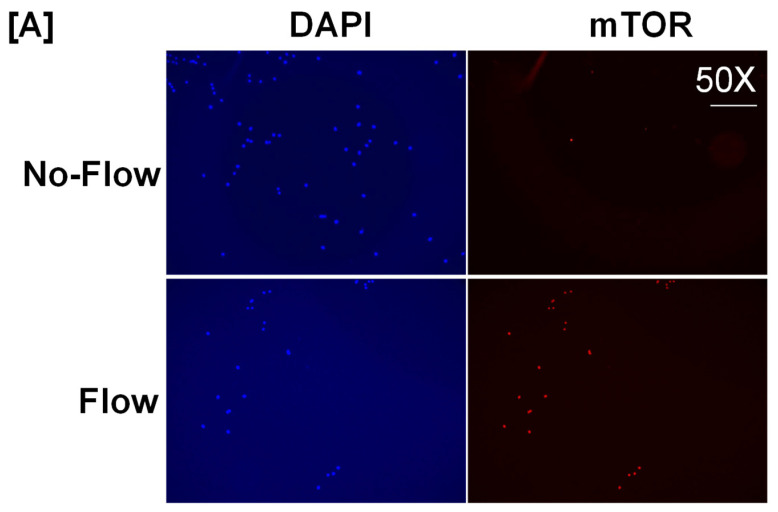

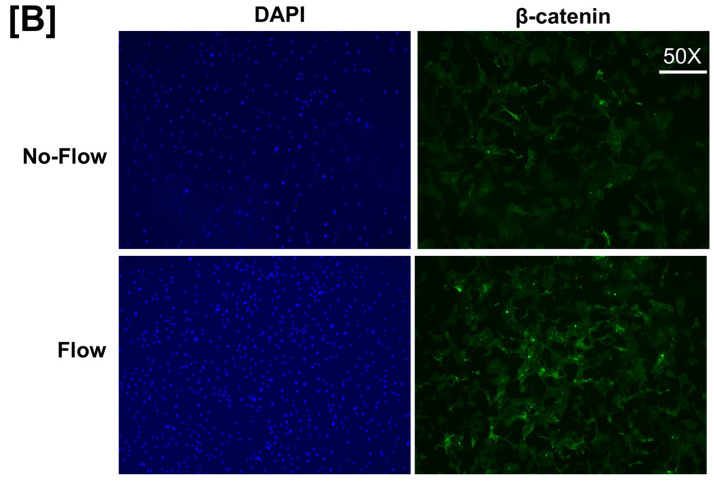
Higher numbers of osteoblasts expressed detectable levels of (**A**) mTOR and (**B**) β-catenin after 30 min of orbital flow compared to No-flow. Red color represents mTOR expression, green represents β-catenin, and blue corresponds to nuclei stained with DAPI. n = 3.

**Figure 5:**
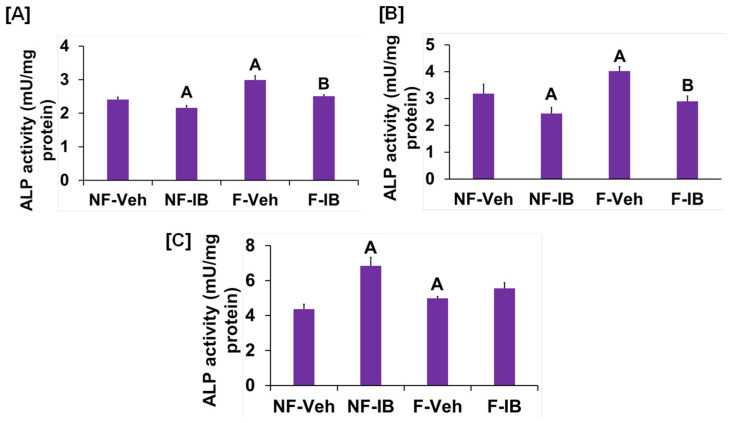
Effects of (**A**) WNT, (**B**) mTOR, and (**C**) JNK signaling inhibitors on orbital flow-induced osteoblast differentiation. NF, No-flow; F, Flow; Veh, vehicle; IB, inhibitor. ^A^ *p* < 0.05 vs. NF-Veh, ^B^ *p* < 0.05 vs. F-Veh. Values are mean ± SEM, n = 5.

It is well known from previous studies that integrins and receptors on the surface of bone cells convert mechanical signals into biochemical signals, which stimulates different cellular processes that contribute to bone formation [23,24]. In this regard, studies using in vitro and in vivo models reported that some of the key signaling pathways (WNT, BMP, mTOR, and JNK) respond to mechanical strain [25,26,27,28]. Therefore, we assessed if these signaling pathways were activated in response to orbital flow. mTOR protein expression was evaluated using immunofluorescent antibodies 30 min after orbital shaking. Figure 4A shows mTOR positive cells, represented by a red dot, in the flow and no-flow groups. We found that there were three times more mTOR positive osteoblasts (16.3 ± 4.4 vs. 5 ± 1.73, *p* = 0.07, n = 3) in the flow group compared to the no-flow group. In addition to mTOR, we also tested if β-catenin expression was stimulated in response to orbital flow, and found that a higher number (15.66 ± 2.6 vs. 6.33 ± 0.33, *p* = 0.02, n = 3) of osteoblasts expressed detectable levels of β-catenin, as represented by the green signal, 30 min post flow compared to no-flow (Figure 4B). The consequences of blocking specific pathways using specific inhibitors on osteoblast differentiation were evaluated, and it was found that blocking mTOR signaling with inhibitors significantly reduced orbital flow-induced ALP activity compared to vehicle-treated controls (Figure 5A). A similar finding was also observed after blocking the WNT signaling pathway (Figure 5B). In contrast, JNK signaling inhibitors did not significantly affect flow-induced osteoblast differentiation (Figure 5C). These data demonstrate that the orbital shaker, like other established in vitro mechanical loading models, stimulated intra-cellular anabolic signaling pathways in response to flow.

## 4. Conclusions

Our findings demonstrate that the flow produced by orbital shaking at a lower frequency is a useful inexpensive model for studying the molecular pathways mediating mechanical strain effects in primary cultures of osteoblasts in vitro.

## Data Availability

Raw data are available up on request.

## Supplementary Materials

The following supporting information can be downloaded at: https://www.mdpi.com/article/10.3390/biology13090646/s1, Supplemental File S1. Video showing orbital shaker used in this study. Supplemental File S2. Video showing see-saw shaker used in this study.
