# Supplementary material for: An In Vitro Orbital Flow Model to Study Mechanical Loading Effects on Osteoblasts
Source: Biology (Basel). 2024 Aug 23;13(9):646. doi: 10.3390/biology13090646 (PMC11428209; doi:10.3390/biology13090646)
Supplement: Supplementary file 1 [file biology-13-00646-s001.zip › biology-3146413-supplementary.pptx]

## Slide 1
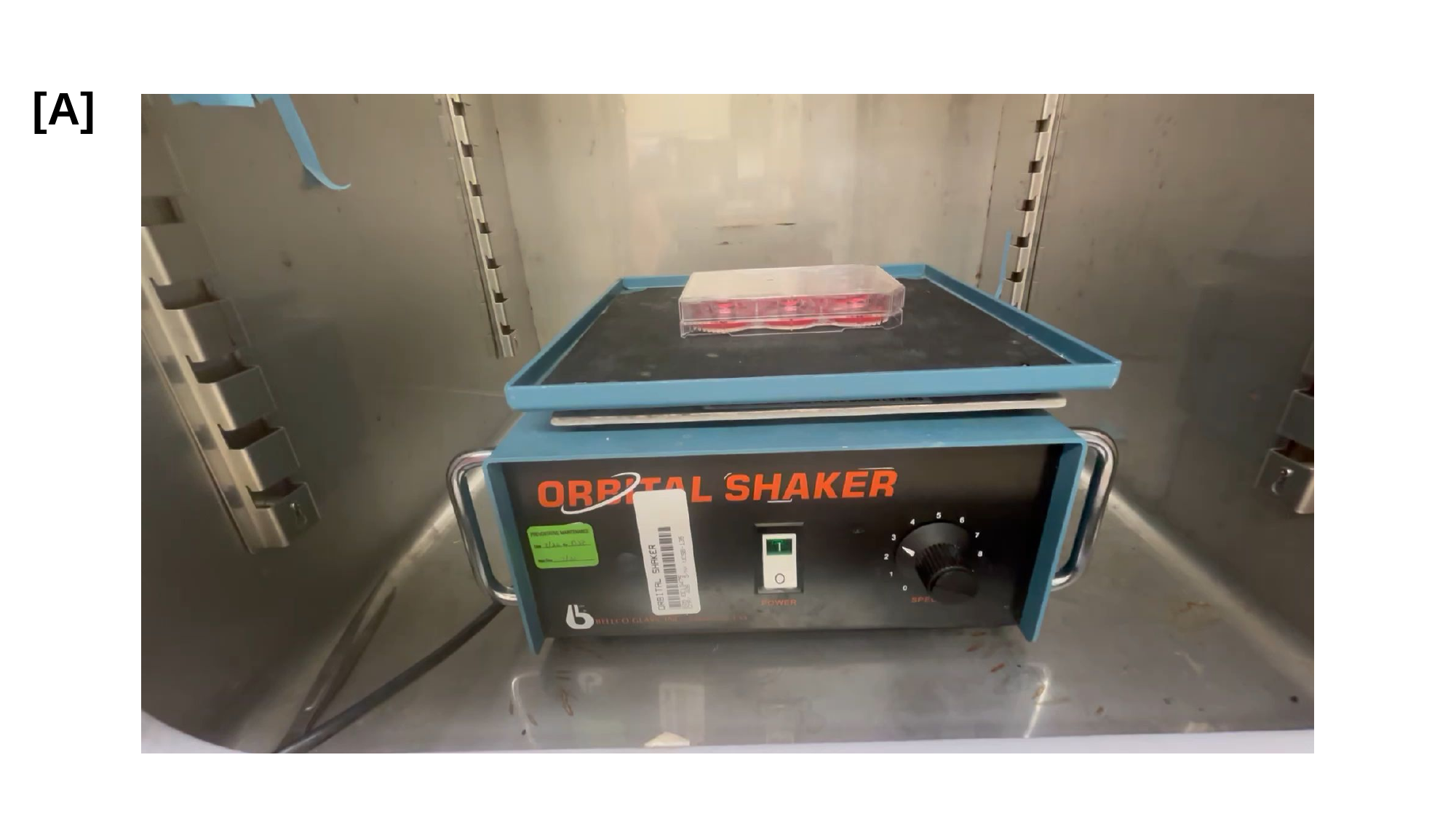

[A]

## Slide 2
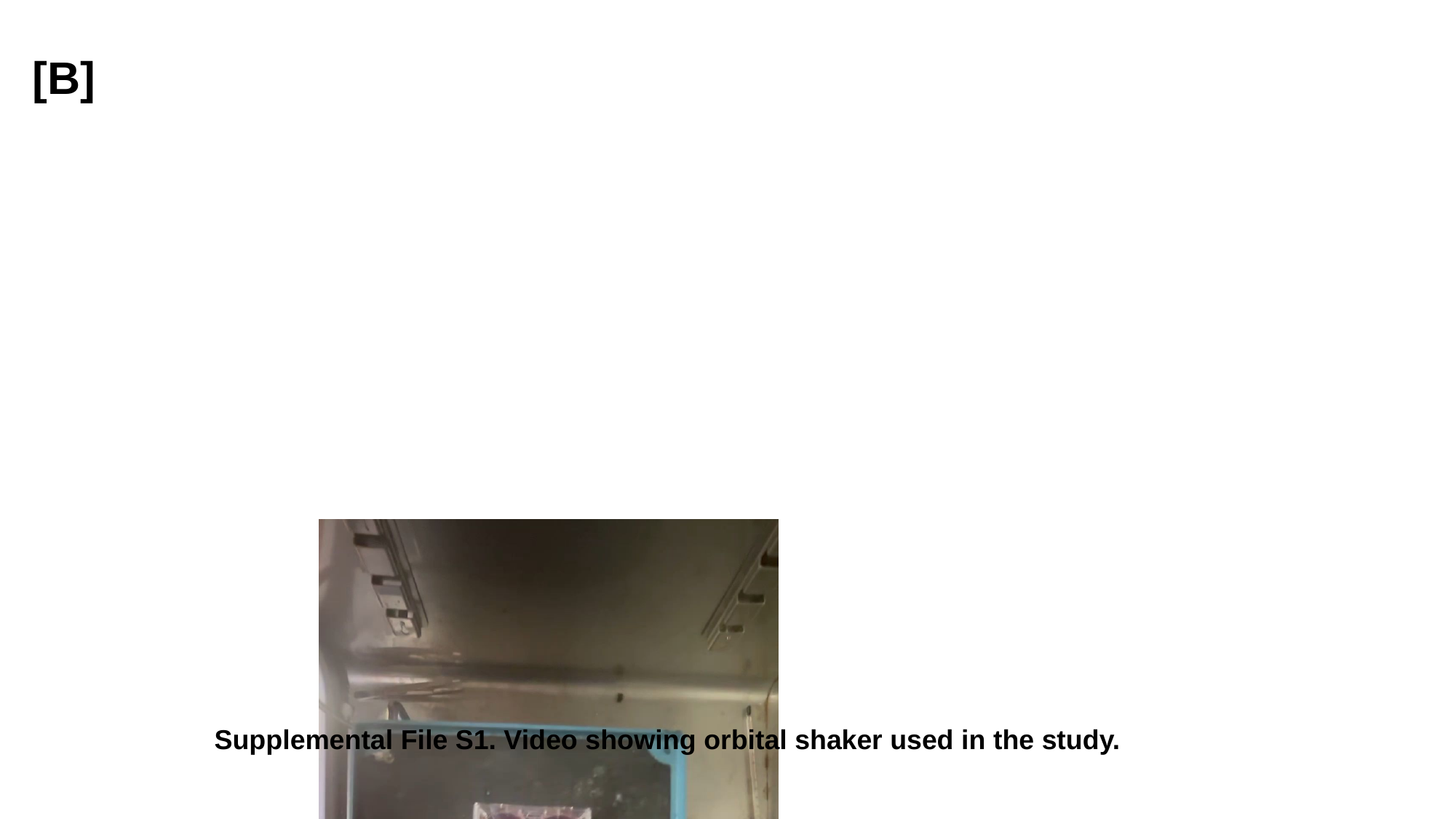

[B]
Supplemental File S1. Video showing orbital shaker used in the study.

## Slide 3
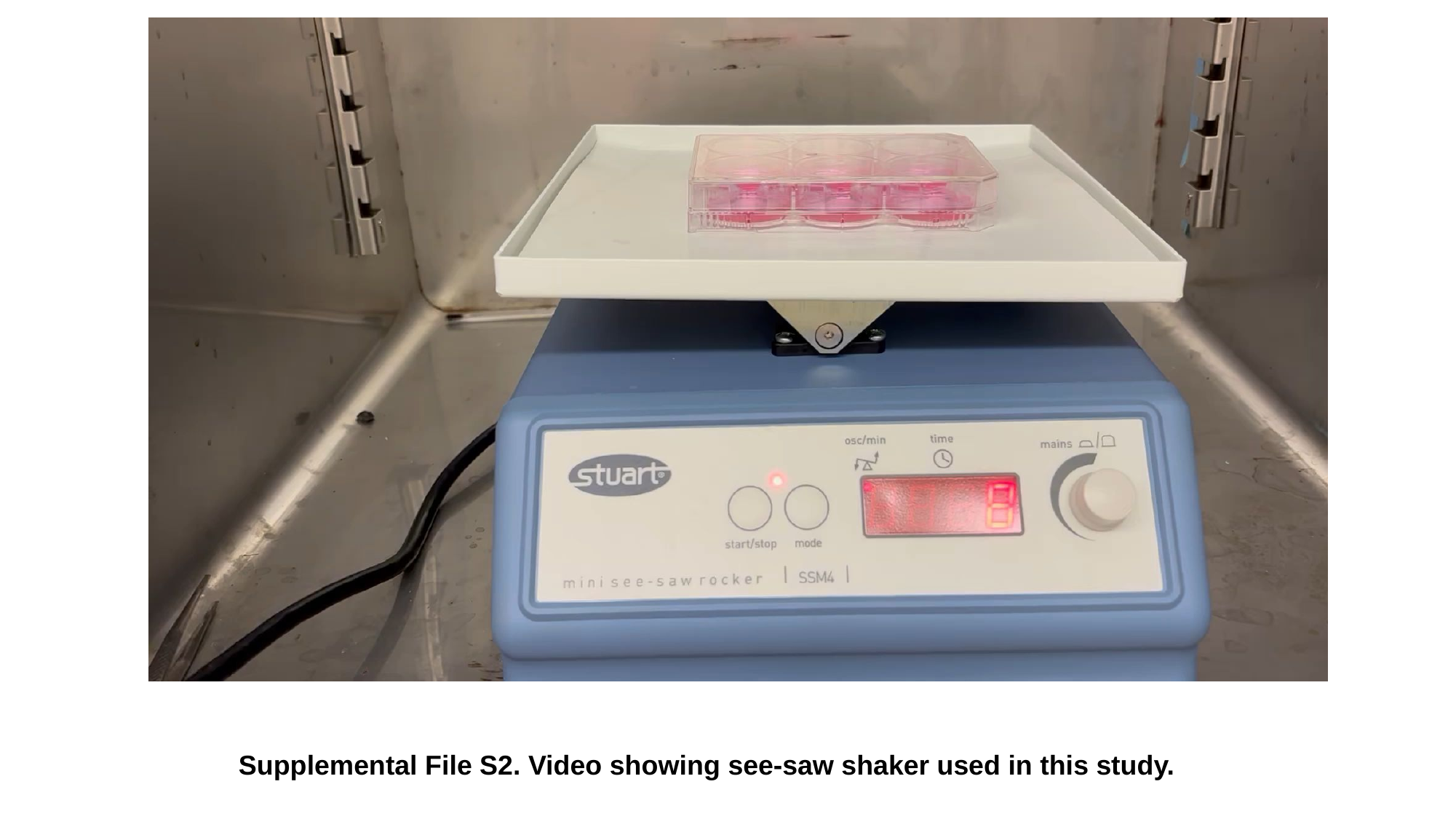

Supplemental File S2. Video showing see-saw shaker used in this study.
